# Remembering Arthur: A Tale of Resilience in Malawi

**DOI:** 10.4269/ajtmh.18-0176

**Published:** 2018-09

**Authors:** Mark Lieber

**Affiliations:** University of California, Irvine School of Medicine, Irvine, California

Arthur Chidote, my first local friend in Malawi, affectionately referred to the most important woman in his life, his grandmother, as “Granny.” I had no idea during my first few weeks in the country that I would eventually meet “Granny” at Arthur’s funeral later that year.

I first met Arthur while working with Partners In Health (PIH) in the remote district of Neno, Malawi, in 2012. Arthur and I were the same age and held the same position, but at different PIH hospitals. He was, in a way, my Malawian doppelgänger.

Our job consisted primarily of tracking medications in our respective hospitals and maintaining medical equipment such as oxygen cylinders, nebulizers, and X-ray machines. We did a lot of the behind-the-scenes work to keep our PIH hospitals stocked and functional.

During my first week in Malawi, Arthur taught me about the four gas cylinders that supplied oxygen to the patients in the adult and pediatric wards. Standing by the side of a young boy with cerebral malaria who was breathing supplemental oxygen through a nasal cannula, Arthur explained to me the importance of keeping the cylinders in stock.

“At a flow rate of 10 L/minute, the cylinder will only last about an hour,” Arthur informed me. “It’s important to have another filled cylinder nearby if you know you’ll need more.”

A few hours later, the young patient with cerebral malaria died. At the time, I was in one of the warehouses trying to find the intravenous quinine that could have saved his life.

Arthur and I spent the next few days rearranging the PIH warehouse to make it easier to find the most important drugs and medical equipment. Eventually, we stumbled upon the donations of intravenous quinine.

My heart sank. “If only I had known where this was a few days ago,” I thought to myself. “Maybe that boy would still be alive.” I confessed to Arthur that I still felt guilty that I had not found the quinine in time, and I blamed myself for the boy’s death.

He sat me down and looked me square in the eye. I could tell that he was about to tell me something potentially life-changing.

“Challenges like this are part of everyday life in Malawi,” he said. The next time a boy with cerebral malaria is admitted to the hospital, he told me, I would know exactly where to go to find the quinine that would save his life.

Maybe the boy’s death had not been in vain, I thought. Somehow, that made me feel a little bit better.

This is only one of many lessons Arthur taught me in our time together.

Like many of the PIH employees in Malawi, Arthur had previously been a patient at the Neno District Hospital—and like nearly three-quarters of the patients at the hospital, Arthur was also HIV positive. When I first arrived in Malawi in 2012, the HIV prevalence in Malawi was close to 15%, and approximately 75% of the country’s hospital beds were filled with people fighting HIV-related coinfections.

Infectious diseases such as malaria, schistosomiasis, and tuberculosis were also highly prevalent in the region, and associated health outcomes were often made worse by the immunodeficiency caused by HIV/AIDS. Because of these factors, as well as a high maternal mortality rate, a worsening food insecurity situation, and inadequate health-care infrastructure and transportation, the average life expectancy in the country hovered around 50 years of age.

About 6 months into my stay in Malawi, my supervisor called to let me know that Arthur had died unexpectedly because of an HIV-related infection.

I was shocked. I had never lost a good friend before. And while I knew that Arthur was HIV positive, I assumed that he was managing the virus with medications. He never seemed sick to me at all.

News of his passing forced me to contemplate the many inequalities that separated his fate from mine. That easily could have been me, I thought, had I been born in a different country or at a different time.

A few days later, a small group of coworkers and I drove to Arthur’s mother’s house for the funeral. The sound of women’s voices wailing echoed onto the street outside the compound, reverberating in our heads as we walked in. The wailing was loudest at the beginning of the ceremony and then later, during the burial. The sounds reminded me of the wailing that we often heard at the hospital whenever a patient died. They were sounds of pain, but they were not just meant for human ears—they also seemed to call to other-worldly spirits.

The funeral began with a traditional lunch of goat, beans, and rice at Arthur’s family’s house. After the meal, the men and women were divided into two different groups. The funeral leader then called out different groups of people from Arthur’s life, such as primary school friends, PIH coworkers, and family members. When the leader called each group, members would stand and one person would give a speech about Arthur. When PIH was called, one of our coworkers gave a touching speech about Arthur’s contagious laugh and his passion for living.

After all of the groups had been called, about eight women then lifted the coffin and began the long, 2-km walk to the cemetery. Whereas I had lost my first close friend in Malawi, Arthur’s mother had lost three children in the past year alone. I have never had children, but I could only imagine the pain that she was feeling at that moment.

In Malawi, approximately one of every five people dies before the age of 18. I tried to picture losing close to 20% of my high school graduating class before adulthood.

I saw death and fear of death daily in the PIH hospital where I worked. Adolescent girls who would come into the maternity ward to give birth were often visibly scared, and understandably so. One in every 36 pregnancies in Malawi ends in maternal death, and 20% of those who die are adolescents. Compared with their adult counterparts, pregnant adolescents are more likely to experience obstructed labor, perinatal sepsis, and hypertensive disorders such as eclampsia and preeclampsia.

Although these patients struck a chord in my heart—and I wanted to do everything I could to help them—they were still a little distant from me. Arthur was not. In him I saw a good friend and a part of myself. Arthur’s friendship and death also taught me that death from treatable causes is not simply tragic. It truly robs the community of wonderful people and steals away hope for a better future.

The weekend following Arthur’s funeral, I attended the wedding of one of our Malawian PIH doctors. The mental shift from funeral to wedding was admittedly jarring. At the same time, I think Arthur would have approved: it seemed a fitting metaphor for how quickly life can vacillate between our highest “ups” and lowest “downs.”

